# Kentucky School for the Instruction of the Blind

**Published:** 1842-03

**Authors:** 


### KENTUCKY SCHOOL FOR THE INSTRUCTION OF THE BLIND.

Last winter, when delivering some popular lectures on Physiology, in the Hall of the Medical Institute, we took occasion to explain the different modes of teaching the blind; and urged the necessity for a school in this State. Mr. Bullock, a representative from this city, soon after introduced into the Legislature, a bill for that purpose, which passed one branch, but was laid over in the other. This winter, Mr. Bullock again brought up the subject, and at the request of Mr. Patton of this city, Dr. Howe of Boston came out with one of his pupils, and Mr. Chapin, the Superintendent of the Ohio School, came from Columbus, with three of the pupils of that institution. At Frankfort they made two exhibitions, with the effect of securing the passage of the bill, with an appropriation conditioned on the contribution of a sum of money by the people of Louisville. In this city, they made several exhibitions, and gave an impulse to the work of contribution. Thus, there is much probability, that within the present year, this interesting institution will be opened. We hope, however, that our medical friends will not relax in their efforts to prevent blindness, in consequence of reading this paragraph; and that none of them will advise parents to procure glass eyes for their children, instead of sending them here when the school shall be opened.

